# Life outcomes in adults living with FASD in a rural South African community: A follow-up study

**DOI:** 10.4102/ajod.v13i0.1386

**Published:** 2024-08-26

**Authors:** Mandi Broodryk, Jaco G. Louw, Debbie Acker, Denis L. Viljoen, Leana Olivier

**Affiliations:** 1 Foundation for Alcohol Related Research, Cape Town, South Africa; 2 Department of Obstetrics and Gynaecology, Faculty of Medicine and Health Sciences, Stellenbosch University, Stellenbosch, South Africa

**Keywords:** prenatal alcohol exposure, persons with disabilities, life course research, fetal alcohol spectrum disorder, fetal alcohol syndrome, fas

## Abstract

**Background:**

Even though adults with foetal alcohol spectrum disorder (FASD) are at risk of negative life outcomes, there is no published evidence of this in South Africa, which has the highest estimated FASD prevalence rate globally.

**Objectives:**

The purpose of the study was to describe and compare the life outcomes of adults with FASD and adults without FASD in a South African rural community, 16 years after diagnosis.

**Method:**

Participants were examined and interviewed regarding their biographical information, knowledge of FASD, information on their family, relationships, home circumstances, education, work and medical history.

**Results:**

Adults with FASD were less likely to be in a relationship and more likely to have poor educational outcomes and to be exposed to violence as victim or perpetrator than their peers who did not have FASD. None of the participants with FASD completed secondary school successfully. No differences were found for independent living, employment, health, substance use and legal outcomes, between the foetal alcohol syndrome (FAS) or partial foetal alcohol syndrome (PFAS) and control group.

**Conclusion:**

While significant differences existed in certain aspects, differences are not as stark as one would expect between individuals with FASD and controls.

**Contribution:**

This study highlights the importance of considering the social context in which a FASD diagnosis is made. The comparative negative impact of an FASD diagnosis and the associated challenges on life outcomes may be less pronounced in rural communities where everyone has fewer opportunities and resources. This can also make the unique needs of persons with disabilities less visible.

## Introduction

Foetal alcohol spectrum disorder (FASD) is an umbrella term for a highly prevalent and preventable spectrum of disorders caused by prenatal alcohol exposure. Foetal alcohol spectrum disorder consists of diagnoses of foetal alcohol syndrome (FAS), partial foetal alcohol syndrome (PFAS), alcohol-related neurodevelopmental disorder (ARND) and alcohol-related birth defects (ARBD). Characteristics of FASD include recognisable facial differences, intrauterine growth restriction or failure to thrive and deficits in neuropsychological development (Hoyme et al. 2016), which all have significant lifelong effects on mental and physical health, behaviour, educational achievements, employment, independent living, substance use and offending behaviour (Domeij et al. 2018; Landgren et al. 2019; McLachlan et al. 2020; Popova et al. 2021; Rangmar et al. 2015; Spohr & Steinhausen 2008; Temple et al. 2021). When referring to the sequalae related to an FASD diagnosis, a distinction between primary and secondary disabilities can be made. Primary disabilities refer to cognitive impairment in individuals with FASD. These disabilities can be measured by general intelligence, mastery of reading, spelling, math, and level of adaptive functioning, which represent the central nervous system consequences of the disorder (Streissguth et al. 1996).

Secondary disabilities refer to problems that occur as a result of primary disabilities and are not present at birth (Moore & Riley 2015; Streissguth et al. 1996). These can be prevented or improved with appropriate interventions and understanding (Streissguth et al. 1996). Previous research on secondary disabilities in FASD populations identified that conflict with the law (Kambeitz et al. 2019; McLachlan et al. 2019; Popova et al. 2021; Streissguth et al. 1996), mental health issues (Grant & Connor 2005; Huggins et al. 2008; Landgren et al. 2019; Pei et al. 2011; Popova et al. 2021; Spohr, Willms & Steinhausen 2007; Temple et al. 2021), alcohol and substance abuse (McLachlan et al. 2020; Streissguth et al. 1996, 2004), physical, sexual or verbal abuse (Kambeitz et al. 2019), educational achievement (Domeij et al. 2018; McLachlan et al. 2020; Streissguth et al. 1996, 2004) and foster or residential care placement (Kambeitz et al. 2019; McLachlan et al. 2020) is common. There is little information about the challenges and life outcomes in adult individuals with FASD compared to those without FASD. Previous literature on this topic focuses on individuals with FASD, which often include individuals with an FAS-specific diagnosis (Grant & Connor 2005; Huggins et al. 2008; Streissguth et al. 1996), without a comparison group. There is also little literature about long-term follow up of an FASD cohort with a focus on life outcomes, especially in comparison to individuals without FASD. Furthermore, research on this topic is lacking in South Africa, where the highest published prevalence rates of FASD have been reported globally. To date, the highest reported FASD prevalence in the world, has been estimated in the Western Cape Province of South Africa, at 310 per 1000 (31%) (May et al. 2022).

This study aimed to describe and compare the life outcomes of adults diagnosed with FASD in a 2001 prevalence study in the Emthanjeni Local Municipality in the Northern Cape Province of South Africa where a prevalence rate of 119.4 per 1000 (11.9%) was reported (Olivier 2017; Urban et al. 2008). We hypothesised that adults with FASD, which included a FAS or PFAS diagnosis specifically, would be less likely to complete secondary education (12th grade), less likely to be employed, more likely to have conflict with the law, have a higher incidence of health problems, less able to live independently, as well as experience more social problems. We will refer to individuals diagnosed with FAS or PFAS instead of FASD as during the prevalence study when the diagnoses were made, ARND and ARBD were not diagnosed. We therefore aim to provide contextual evidence that will address the presumptions made in our hypothesis and the gaps in current published evidence.

## Research methods and design

### Community

The community where this study was conducted and from which the study participants came, is close to the centre of South Africa, 250 kilometres from Kimberley, the capital of the Northern Cape Province. This rural town is part of the Emthanjeni local municipality which, at the time of this follow-up study, had a population size of approximately 47 609 (Northern Cape Provincial Treasury 2019). An unemployment rate of 35.10% has been reported with only 4% of the population having completed secondary school (12th grade) (Northern Cape Provincial Treasury 2019).

### Sample

This was a longitudinal cohort study. This study followed up on a cohort of individuals diagnosed with FASD as part of an FASD prevalence study conducted on the Emthanjeni municipality in 2001, as well as their matched controls (Urban et al. 2008). In the 2001 study, children diagnosed with FASD were matched with healthy controls based on age and sex (Urban et al. 2008). All cases and controls from the 2001 study were invited to participate and no further matching of controls was performed.

The individuals who were able to locate and who still stayed in the same town in the Northern Cape, and provided informed consent, participated in this study. All participants stayed in areas commonly associated with low socioeconomic status, and they attended school in the same areas. As the controls were matched in 2001 and as they lived in the same environment there were no significant demographic or social differences. In the 2001 study, no significant differences in socioeconomic conditions were reported except for a higher occurrence of part-time employment or unemployment for the guardians of the children in the FASD group (77% in the control group compared to 92% in the FASD group). This data included two study sites, however, and a specific comparison for the current setting is not available.

### Ethical considerations

Ethical approval for this study was obtained from the Faculty of Medicine and Health Sciences’ Health Research Ethics Committee at Stellenbosch University (Ethics reference number: N13/01/008A). Written informed consent was obtained from 30 of the participants who were diagnosed in 2001 and 30 controls, and the study was conducted in accordance with the Helsinki Declaration as revised in 2013.

### Data collection

Community workers located participants from the earlier study still residing in the area, made possible by the small size of the community. Each potential participant was then approached, and the study procedure, aims, expectations, risks and benefits associated with participation were discussed. Written informed consent was obtained from participants. Participants were first examined by a clinician and then interviewed by trained interviewers about their biographical information, understanding of FASD, information about their family, relationships, home circumstances, education, work and medical history, as well as risk-taking behaviour. A parent, guardian or collateral informant (in cases where the parents were deceased or untraceable) of each participant was also interviewed about the participant. Questions that were asked were based on previous literature on negative life outcomes and epidemiology studies (Chudley et al. 2007; Moore & Riley 2015). Further input was also obtained from professionals working in the area of expertise to inform the information collected.

### Data analysis

Descriptive statistics were used to analyse information obtained on the participants’ life outcomes from the interviews conducted. To establish whether differences between group membership (FAS or PFAS vs. Control) and specific life outcomes (yes or no) were present, Pearson chi-square analyses were conducted for each life outcome measure. As both variables were categorical, frequency data were used. In addition, if the sample was large, and the expected count per cell was more than five, the assumptions to use the chi-square test statistic were met. Where significant differences were found, the strength of the correlation was calculated using the Cramer’s V test (McHugh 2013). If the sample size was too small and less than 80% of expected counts were below 5, the Fisher’s exact test (2-sided) was used. If significant, the odds ratio was used as a risk measure, and the Cramer’s V test was calculated as an effect size. Study data were captured and managed using REDCap electronic data capture tools (Harris et al. 2019). The study data were analysed using IBM SPSS Statistics (Version 25) (‘SPSS Statistics for Windows’ 2017).

## Results

### Demographic data

In total, 52 participants completed the study with an equal number of females and males (*n* = 26; 50%). See Table 1 for a summary of the age and sex for each group (FAS or PFAS vs. Controls). Most of the participants’ home language was Afrikaans (*n* = 46, 88.5%), followed by bilingual (not specified) (*n* = 2, 3.8%), Xhosa (*n* = 2, 3.8%), English (*n* = 1, 1.9%) and other (not specified) (*n* = 1, 1.9%).

**TABLE 1:** Age and sex at baseline and follow up by group.

Age and sex	FAS or PFAS					Control					Total				
	%	*n*	*M*	s.d.	Range	%	*n*	*M*	s.d.	Range	%	*n*	*M*	s.d.	Range
Age (baseline)	-	-	6.90	0.99	6–10	-	-	6.79	0.55	6–7	-	-	6.84	0.78	6–10
Age (follow-up)	-	-	23.30	2.36	22–33	-	-	22.59	0.50	22–23	-	-	22.9	1.64	22–33
Female	48	11	-	-	-	51	15	-	-	-	50	26	-	-	-
Male	52	12	-	-	-	49	14	-	-	-	50	26	-	-	-

FAS, foetal alcohol syndrome; PFAS, partial foetal alcohol syndrome; s.d., standard deviation; M, mean.

### Life outcomes

#### Household and social

After Pearson chi-square analyses were conducted on the life outcome measures (binary yes or no answers), a significant difference between the group that the participant belonged to and whether they were currently in a relationship at the time of the interview was found, *X*^*2*^ (1, *N* = 52) = 4.461, *p* = 0.035. However, the effect size for this finding, Cramer’s *V*, was low, φ = 0.293 (Cohen 1992). Seventy-two per cent of the controls had a partner with only 43.5% in the FAS or PFAS group reporting the same. No association between groups and whether participants had children was found, *X*^*2*^ (1, *N* = 51) = 2.48, *p* = 0.115. A significant association between groups and whether both participants’ parents were still alive was found, *X*^*2*^ (1, *N* = 52) = 4.461, *p* = 0.0.35. However, a low effect size was found, φ = 0.293. Sixty-seven participants in the control group had both parents still living in comparison with 32% in the FAS or PFAS group. Refer to Table 2.

**TABLE 2:** Life outcome comparisons between groups.

Life outcomes	Control		FAS or PFAS		*p*
	*n*	%	*n*	%	
**Household and social**					
In a relationship	21	72.41	10	43.48	0.035*
Has children	17	58.62	8	34.78	0.115
Experienced partner violence in current relationship	1	3.45	3	13.04	0.117
Grew up with					
Biological mother or father	17	58.62	10	43.48	-
Maternal grandparents	5	17.24	3	13.04	-
Paternal grandparents	0	0.00	1	4.35	-
Siblings	2	6.90	1	4.35	-
Extended family	2	6.90	0	0.00	-
‘Other’	3	10.34	7	30.43	-
Foster care	0	0.00	1	4.35	-
Both parents are still alive	21	72.41	10	43.48	0.035*
**Parent(s) who have passed away**					
Mother	2	6.90	2	8.70	-
Father	4	13.79	3	13.04	-
Both parents	1	3.45	6	26.09	-
**Who do you currently live with?**					
Live alone	1	3.45	0	0.00	-
Partner/spouse	2	6.90	1	4.35	-
Parent or parents	10	34.48	2	8.70	-
Sibling	1	3.45	2	8.70	-
Extended family	8	27.59	6	26.09	-
Other	1	3.45	6	26.09	-
More than one specified	6	20.69	7	30.43	-
Foster parent(s)	0	0.00	3	13.04	-
Own child	0	0.00	1	4.35	-
Has been homeless before	4	13.79	2	8.70	0.568
**Independent living outcomes**					
Currently in control of own finances	21	72.41	17	73.91	0.904
Can live alone without help in the household	25	86.21	17	73.91	0.264
**Educational outcomes**					
Completed secondary school	16	55.17	0	0.00	< 0.01*
Studied after secondary school	6	20.69	0	0.00	0.030*
Ever repeated a grade	15	51.72	18	78.26	0.048*
Missed school regularly	3	10.34	9	39.13	0.014*
Ever attended a special needs class or LSEN† school	4	13.79	9	39.13	0.036*
Ever had extra support at school	1	3.45	4	17.39	0.090
**Employment and income**					
Unemployed	26	89.66	20	86.96	0.762
Additional income					
Disability grant	0	0.00	2	8.70	-
Child support grant	5	17.24	5	21.74	-
Child maintenance	2	6.90	0	0.00	-
Care dependency	0	0.00	1	4.35	-
**Legal/conflict with the law**					
Ever been stabbed or shot	2	6.90	10	43.48	0.002*
Ever stabbed or shot someone else	0	0.00	5	21.74	0.007*
Ever been in trouble with the police or law	6	20.69	5	21.74	0.979
Ever spent time in jail	3	10.34	4	17.39	0.303
Currently belonging to a gang	1	3.45	1	4.35	0.887

Note:

* *p* < 0.05;

† , LSEN = Learners with Special Educational Needs.

FAS, foetal alcohol syndrome; PFAS, partial foetal alcohol syndrome.

#### Independent living

No significant association was found between groups and whether a participant manages their own finances, *X*^*2*^ (1, *N* = 52) = 0.015, *p* = 0.904. No significant association between groups and the ability to function independently was found, *X*^*2*^ (1, *N* = 52) = 1.248, *p* = 0.307). Refer to Table 2.

#### Education

With regard to schooling, a significant association between groups and highest level of education was found, *X*^*2*^ (1, *N* = 52) = 10.199, *p* = 0.002. All the participants in the control group reported their highest qualification was in secondary school (8th to 12th grade in South Africa), with only 70% of participants in the FAS or PFAS group reporting the same. A significant association between group and completing their full secondary school education was also found, *X*^*2*^ (1, *N* = 52) = 18.330, *p* = 0.000, with a high effect size φ = 0.594. None of the participants in the FAS or PFAS group successfully completed secondary school, with 55% of participants in the control group completing secondary school successfully. A significant difference was found for group membership and repeating a grade *X*^*2*^ (1, *N* = 52) = 3.895, *p* = 0.048, with a low effect size, φ = 0.048. Just over half of the control group reported repeating at least one grade, compared to 78% of the FAS or PFAS group, with a significant association, *X*^*2*^ (1, *N* = 52) = 5.987, *p* = 0.014, and a moderate effect size, φ = 0.339. Ten per cent of the control group missed school regularly compared to 39% of the FAS or PFAS group. A significant association between group membership and attending a special needs class at school or attending a Learners with Special Educational Needs (LSEN) school was found, *X*^*2*^ (1, *N* = 52) = 4.392, *p* = 0.036, with a low effect size of φ = 0.291. Fourteen per cent of the control group attended these classes or schools compared to 39% in the FAS or PFAS group (refer to Table 2).

#### Employment and income

Group and employment status did not show a significant association, *X*^*2*^ (1, *N* = 52) = 0.092, *p* = 0.762. with majority of the total sample reported being unemployed (*n* = 46, 88.46%). No associations were found between groups for receiving social grants, *X*^*2*^ (1, *N* = 44) = 0.052, *p* = 1.000, with only 36.36% of the total sample receiving a social grant, regardless of the high unemployment rate for the total sample (64%). No significant associations were found between groups and having experienced times where participants could not afford to eat for the day, *X*^*2*^ (1, *N* = 51) = 2.021, *p* = 0.155, with 56.86% (*n* = 28) of participants in the total sample reporting that they did experience this.

#### Legal and/or conflict with the law

A significant association between groups and having been stabbed or shot before was found, *X*^*2*^ (1, *N* = 52) = 9.670, *p* = 0.002, with a moderate effect size, φ = 0.431. Forty-three per cent of the FAS or PFAS group were stabbed or shot before, with 6% of the control group reporting the same. In addition, a significant difference was found for group membership and having stabbed or shot someone else before, *X*^*2*^ (1, *N* = 51) = 7.307, *p* = 0.011. An odds ratio was calculated as the Fisher’s exact test statistic was used because of 50% of expected counts being under 5. The odds ratio was calculated as 1.294, with the control group 1.294 times more likely not to stab or shoot someone in the past. Zero per cent of the control group reported having shot or stabbed someone before compared to 23% for the FAS or PFAS group. Refer to Table 2 and Table 3 for a summary of the non-significant results.

**TABLE 3:** Health behaviour outcome comparisons between groups.

Health behaviour outcome	Control		FAS or PFAS		*p*
	*n*	%	*n*	%	
**Health**					
Ever admitted to the hospital	11	37.93	12	52.17	0.474
Under specialist care for treatment	4	13.79	4	17.39	0.637
Currently on chronic medication for a medical condition	3	10.34	0	0.00	0.112
Any history of experiencing seizures	4	13.79	2	8.70	0.568
Born with birth problems or conditions	2	6.90	6	26.09	0.091
Ever experienced a head injury	3	10.34	2	8.70	0.841
Experience problems with sleeping, tired during the day on most days	3	10.34	6	26.09	0.136
Ever been diagnosed with major depression	1	3.45	0	0.00	0.867
Alcohol and substance use					
Ever used alcohol	26	89.66	19	82.61	0.460
Still consume alcohol	19	65.52	11	47.83	0.763
Currently using tobacco	14	48.28	16	69.57	0.103
**Frequency of tobacco use**					
1–5 per day	9	31.03	8	34.78	-
5–15 per day	4	13.79	4	17.39	-
more than 15 per day	0	0.00	2	8.70	-
less than 1 per day	0	0.00	1	4.35	-
missing	1	3.45	1	4.35	-
Use any drugs	3	10.34	5	21.74	0.250
‘tik’/crystal meth	0	0.00	1	4.35	-
‘dagga’/marijuana	3	10.34	4	17.39	-

FAS, foetal alcohol syndrome; PFAS, partial foetal alcohol syndrome.

## Discussion

This study aimed to compare life outcomes of adults with and without FAS or PFAS. The majority of previous research on individuals with FAS did not have a control group to assess the differences in life outcomes and between peer groups (Freunscht & Feldmann 2011) with a high majority of their sample populations being adoptees or foster care patients or in assistant living environments (Freunscht & Feldmann 2011). Most participants with FAS or PFAS in this study’s cohort (66%) reported growing up with a family member, with 43% of individuals growing up specifically with only their biological mother or father. Individuals with FAS or PFAS were as likely to grow up in the foster care system or with non-relatives as those without FAS or PFAS. Having a diagnosis of FAS or PFAS increased the likelihood of having one or both parents’ deceased and decreased the likelihood to be in a relationship at the time of the study. This could be because of an increased likelihood of parental alcohol abuse and associated adverse physical and psychosocial consequences (Matzopoulos et al. 2014; Probst et al. 2018).

Clear differences between education level were found, with individuals with FAS or PFAS, being more likely to repeat grades, attend special needs classes or schools, miss school regularly and were less likely to complete their secondary school education. This is consistent with a previous study with a Swedish cohort where individuals with FAS were more likely to complete special education and/or primary school as their highest education level (Rangmar et al. 2015). This finding suggests that these individuals struggled to keep up with the available curriculums at school and emphasises the need for tailored support for individuals diagnosed with FAS or PFAS (Millians 2015; Millar et al. 2017). Furthermore, none of those in the FAS or PFAS group in this study successfully completed secondary school compared to 55% of the control group. When comparing this to the Swedish cohort where at least 45.7% of individuals diagnosed with FAS completed secondary education, our finding seems like an especially poor outcome. As only 55% of controls completed secondary school in our sample however, this is not as striking. An important factor contributing to lower academic achievement could be that the participants in our study fall within a low socioeconomic status cohort, which is associated with lower academic success (Chevalère et al. 2023) and the neurodevelopmental challenges faced by individuals in the FAS or PFAS group could further impact academic achievement (Lange et al. 2017). The secondary school dropout rate seems to be reflected in the high unemployment rate of this cohort. The high unemployment rate in the community could be linked to the community’s anecdotal report that low priority is placed on education, which also explains the high dropout rate in the control group. Learners might have little motivation to learn and stay in school because of the low priority placed on education from communities and families (Nortje 2017). Interestingly, no differences were found with regards to independent living, managing one’s own finances, employment status, or likelihood to receive government social relief grants.

This is inconsistent with previous literature in FAS populations (Freunscht & Feldmann 2011). It appears as if the true impact of a FAS or PFAS diagnosis was not necessarily depicted due to environmental factors of this study’s participants. Low socioeconomic status, lack of job opportunities, lack of academic achievement and the high level of unemployment across the entire sample (88%) suggest there might not be opportunities to thrive as they would be in other communities with more resources and access to services. No difference in health-related outcomes and reports of current physical violence or abuse by partners were found. A lack in difference between health-related outcomes is surprising, as individuals with FAS or PFAS have an exceptionally high frequency of adverse health outcomes (Popova et al. 2021) and have been shown to utilise health care services more regularly (Credé et al. 2011). It might be that a general lack of access to healthcare and not going to the local primary healthcare clinic or doctor as often had an impact on this finding. Something like depression will not be diagnosed if the individuals do not seek out treatment or know that they have symptoms of depression due to a lack of awareness or psychoeducation. Consistent with findings from Rangmar et al. (2015), no differences were found in outcomes related to criminal history or trouble with the law. Interesting to note was, that differences between the likelihood of stabbing or shooting someone else or being the victim thereof, were more likely in the FAS or PFAS group. This may allude to problems with aggression, impulse control and not understanding the consequences of actions that are often associated with FAS (Lange et al. 2018). In contrast to previous studies, no differences were found in alcohol and/or substance use behaviours, history of acquiring a head injury, experiencing seizures or birth problems or defects (Rangmar et al. 2015).

## Conclusion

Our findings highlight the differences between adults with FAS or PFAS, and adults without FAS or PFAS in a South African rural community. To the authors’ knowledge, this study is the first of its kind comparing life outcomes and not merely reporting the frequency of these outcomes in a FAS or PFAS population. Adults with FAS or PFAS face specific challenges when compared to controls. However, our findings suggest that individuals with FAS or PFAS not only face their own unique challenges as persons with disabilities, but additionally need to combat the socio-economic challenges that impact all adults living in the same rural community, regardless of a FAS or PFAS diagnosis. In a community where the percentage of unemployment in the total sample is above 85%, with no differences between the persons with disabilities and controls, the needs of adults with FAS or PFAS are not only related to their disability, but also needs to be investigated in the socio-economic setting from a holistic perspective. The poor educational outcomes in the FAS or PFAS group highlight the failures of the education system in supporting persons with disability. More allowance must be made for special schooling, remedial education and the support of learners with special needs in the mainstream. Although this is not unique to persons with FAS or PFAS, we should be concerned about the lack of health and welfare services available to these individuals. They may need long-term care and their subjective evaluation of their ability to live independently cannot be taken at face value. In this rural and under-resourced community, there is no social safety net for persons with FAS or PFAS whose caregivers pass away or can no longer look after them. Policy makers should seriously consider the long-term needs of those affected by FAS or PFAS, especially given the high prevalence rate in this area.
